# Anatomy of Rouviere's Sulcus and Its Association with Complication of Laparoscopic Cholecystectomy

**DOI:** 10.1155/2020/3956070

**Published:** 2020-08-24

**Authors:** Abhijeet Kumar, Rupesh Shah, Narendra Pandit, Suresh Prasad Sah, Rakesh Kumar Gupta

**Affiliations:** ^1^MIS Division, Department of Surgery, BPKIHS, Dharan, Nepal; ^2^Surgical Gastroenterology Division, Department of Surgery, BPKIHS, Dharan, Nepal

## Abstract

**Methods:**

This is a prospective observational study involving patients of age ≥16 years who underwent laparoscopic cholecystectomy for uncomplicated gall stone at BPKIHS between May and July 2019.

**Result:**

230 cases were analyzed, and RS was present in 90.4%. Open sulcus type was the commonest (54%), followed by scar type (22.9%), closed sulcus type (12.5%), and slit type (10.6%), respectively. In 59.1% of cases, it was oblique to the anterior, inferior, and external edge of the liver, while in the remaining cases, it was transverse. The mean ± SD values for operative time and duration of hospital stay in the RS visible and the RS not visible groups were 29.16 ± 8.736 and 42.9 ± 23.646 minutes, and 1.26 ± 0.440 and 1.90 ± 0.910 days, respectively (*p* value ≤0.001). One *minor* complication occurred in each group: RS initially visible group and RS visible on the adhesion release group, while 3 *minor* complications occurred in the RS not visible group. Only one *major* complication occurred in the RS not visible group.

**Conclusion:**

Identification of RS by operating surgeons is a predictor of safe laparoscopic cholecystectomy.

## 1. Introduction

The Rouviere's sulcus (RS) represents a cleft, anterior to the traditionally described “segment 1 of liver” which has been recently described by Couinaud as segment IX [1]. Whatever is previously known about the sulcus came to us from some seminal studies on liver anatomy by Reynaud, Gans, and Couinaud who just noted that this sulcus was present in the majority [1–3].

Hugh et al. were the first to draw attention to its importance during laparoscopic cholecystectomy because it accurately indicates the plane of common bile duct (CBD) [4]. However, if one considers the anatomy of the RS, there are only limited studies in the literature. Its frequency and morphology are not well defined. This study was undertaken to describe the detail anatomy of RS in the population of our part of the world and to discuss the critical aspects of incorporating this useful landmark in safe cholecystectomy.

We believe that this study may increase awareness regarding variations in anatomy of Rouviere's sulcus and guide surgeons to perform safe laparoscopic cholecystectomy.

## 2. Aim

We aim to find the distribution of anatomical characteristics of RS seen during laparoscopic cholecystectomy in the population of our part of the world and its association with bile duct injury and other comorbidities.

## 3. Objectives

### 3.1. Primary Objective

Our primary objective is to measure the distribution of RS (present/absent) and its type (open/close/slit/scar; oblique/transverse) in the population of our part of the world.

### 3.2. Secondary Objective

Our secondary objective is to find the association of RS with incidence of complications (such as bile duct injury, port site infection/hematoma, and others, occurring within 2 weeks of surgery) and other morbidities such as operative time, conversion to open procedure, and duration of hospital stay.

## 4. Materials and Methods

This is a prospective observational study involving humans, with single institution-based setting and purposive sampling technique. The study period was 3 months (from May to July 2019). All patients of age ≥16 years with uncomplicated gall stone disease who underwent laparoscopic cholecystectomy at BPKIHS were included in the study. The exclusion criteria included the following: age <16 years, complicated gall stone disease, patients willing to undergo open cholecystectomy, and being not fit for general anesthesia

### 4.1. Sample Size Calculation

Few studies on similar topics are available in literature. In a study by Dahmane et al., open type Rouviere's sulcus (RS) was found in 70% of livers [5]. Therefore, we calculated the sample size using Cochran's formula (at 95% CI and 90% power) as follows:

(1) $$\begin{array}{c} N=\frac{Z^{2}pq}{L^{2}}, \end{array}$$

where *Z*-value = 1.96 at 95% confidence, *p*=70, *q* = 30, and *L* = 10% of *p*=7.

By adding 20% to the sample size for nonresponse error, a total of 198 patients would have been adequate for the study.

However, during the study period, we encountered 230 patients fulfilling the inclusion criteria, and all were enrolled in the study.

### 4.2. Ethical Clearance

The study was performed in accordance with the principle of the Declaration of Helsinki and was approved by the Institutional Review Board on 26 March 2019.

### 4.3. Conceptual Framework

#### 4.3.1. Enrollment of Patients

All patients of age ≥16 years with uncomplicated gall stone disease who underwent laparoscopic cholecystectomy at BPKIHS from May to July 2019 were enrolled in the study. A detailed clinical history was recorded in a preset proforma. Ultrasonography of abdomen and pelvis and other relevant investigations as per protocol were performed. All patients were screened for exclusion criteria, and if any was present, they were excluded. After informed consent, they were posted for elective laparoscopic cholecystectomy.

Following general anesthesia, port placement, positioning, and proper gallbladder traction, RS whether initially visible to the operating surgeon or not was noted. If RS was not initially visible, the likely reason (such as omental/bowel adhesion, absence of RS, cirrhosis of liver) was noted, and, in case of adhesion, on release of adhesion whether RS became visible before dividing the cystic artery/duct or not was noted. In RS visible group (either initially or following adhesion release), RS characteristics (in terms of its type: open sulcus/closed sulcus/slit/scar; orientation: oblique/horizontal) were noted by the operating surgeon. The dimensions (width and depth) of the RS were measured intracorporeally by comparison with the standard distance between tips of two jaws of dissecting forceps. If the sulcus was deep and wide enough (centimetric), it was described as deep sulcus. Further, a note was made as to whether the deep sulcus was open or closed, depending on whether its medial end was open towards the porta hepatis making the portal structures visible or closed towards the porta hepatis. If the sulcus was too small in its depth and breadth (subcentimetric) so that only length was measurable, it was called slit sulcus. In case of invisible RS even on release of adhesion, the likely reason (such as absence of RS, cirrhosis of liver) was noted. In all cases, complications (recognized intraoperatively or within 2 weeks postoperatively) if any, operative time, and duration of hospital stay were noted. The data was then analyzed.

### 4.4. Operational Definitions

**Figure 1:**
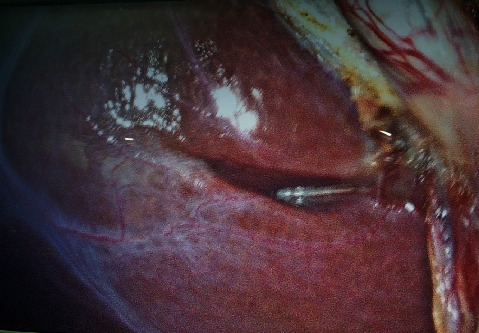
Open type, transverse RS.

**Figure 2:**
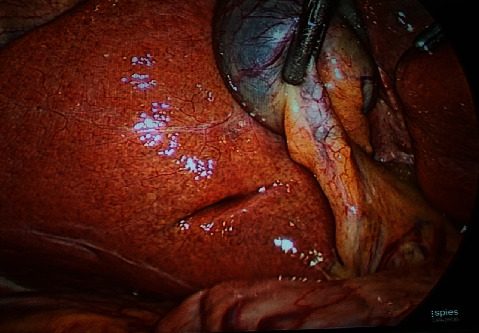
Closed type, oblique RS.

The sulcus was categorized anatomically as either *deep sulcus*, *narrow (slit)*, or *scar*. If the sulcus was deep and wide enough (centimetric), it was described as deep sulcus. Further, a note was made as to whether the deep sulcus was open (Figure 1) or closed (Figure 2), depending on whether its medial end was open towards the porta hepatis making the portal structures visible or closed towards the porta hepatis.

**Figure 3:**
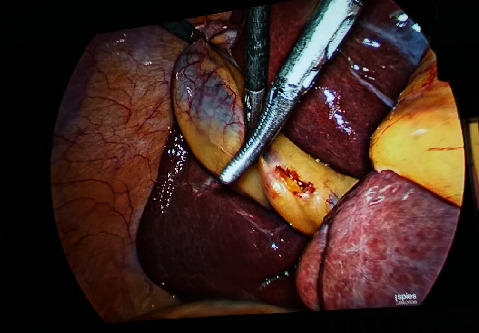
Slit type, oblique RS.

If the sulcus was too small in its depth and breadth (subcentimetric) so that only length was measurable, it was called slit sulcus (Figure 3).

**Figure 4:**
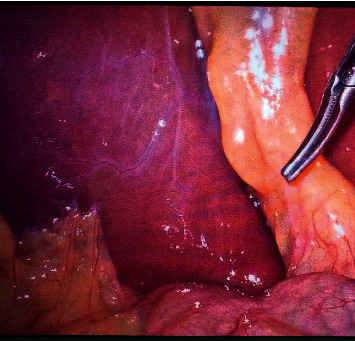
Absence of RS.

Sometimes the sulcus might have just appeared as a scar only and it may be even absent (Figure 4).

We have considered bile duct injury (identified intraoperatively or postoperatively) as *major complication*, while other complications such as port site hematoma, port site SSI, and serous/serosanguinous collection in GB fossa were considered as *minor complications*.

### 4.5. Statistical Analysis

Data was entered in Microsoft Excel and analyzed by SPSS 11. Data was analyzed using descriptive statistics for RS characteristics and patient data, *chi square or Fisher exact test* to compare categorical data, and Student's *t*-test (if data is normally distributed) or Mann–Whitney *U* test (if data is not normally distributed) for continuous data. A *p* value of less than 0.05 was considered statistically significant throughout.

## 5. Results

**Figure 5:**
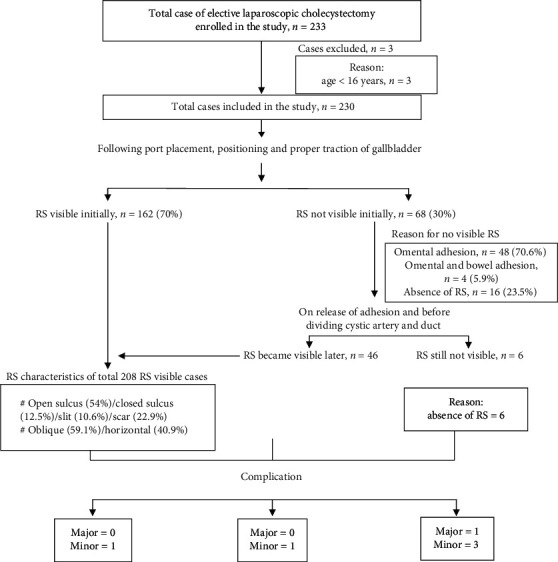
Overall findings of the study.

A total of 233 patients were enrolled in the study. 3 patients were excluded from the study.  Figure 5 illustrates overall findings of the study.

### 5.1. Age Distribution

**Figure 6:**
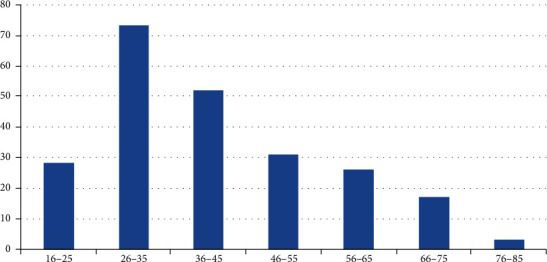
Age distribution.

The age of the patients enrolled in the study ranged from 16 to 80 years with a mean age of 41.56 ± 14.27 years. The majority were in the range of 26–35 years of age (Figure 6).

### 5.2. Gender Distribution

**Figure 7:**
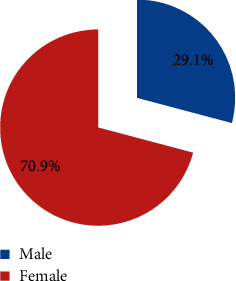
Gender distribution.

In the study, there were 67 (29.1%) males and 163 (70.9%) females with female preponderance of gall stone with ratio of 2.43 : 1 (Figure 7).

### 5.3. Visibility of RS following Port Placement, Positioning, and Proper Traction of Gallbladder

Following port placement, positioning, and proper traction of gallbladder, RS was initially visible to the operating surgeons in 162 (70%) cases, and in 68 (30%) cases, RS was not initially visible. The reasons for initial invisibility of RS were omental adhesion in 48 (70.6%) cases, absence of RS in 16 (23.5%) cases, and omentum-bowel adhesion in 4 (5.9%) cases. Out of 52 cases of initially invisible RS due to adhesion, on release of adhesion in 46 cases, RS became visible to the operating surgeon before dividing cystic artery/duct, while in 6 cases, RS was not visible because of its absence.

### 5.4. RS Characteristics

RS was present in 208 (90.4%) cases, of which open sulcus type, closed sulcus type, slit type, and scar type RS were identified in 114 (54.0%), 26 (12.5%), 22 (10.6%), and 46 (22.9%) cases, respectively.

In 123 (59.1%) cases, it was oblique to the anterior, inferior, and external edge of the liver, and in 85 (40.9%) cases, it was transverse.

### 5.5. Operative Time and RS

The mean ± SD values for operative time in the RS visible and the RS not visible groups were 29.16 ± 8.736 and 42.9 ± 23.646 minutes, respectively (*p* value ≤0.001) (Table 1).

### 5.6. Duration of Hospital Stay and RS

The mean ± SD values for duration of hospital stay in the RS visible and the RS not visible groups were 1.26 ± 0.440 and 1.90 ± 0.910 days, respectively (*p* value ≤0.001) (Table 1).

### 5.7. Intra/Postoperative Complications and RS

Only 2 cases in RS visible before dividing cystic artery/duct group developed complications (port site hematoma: 1; serosanguineous collection in GB fossa: 1) as shown in Table 2. Port site hematoma was managed by evacuation, and serosanguineous collection in GB fossa was managed by image guided aspiration under antibiotic coverage.

4 cases in RS not visible before dividing cystic artery/duct group developed complications (port site hematoma: 1; port site SSI: 1; serosanguineous collection in GB fossa: 1; bile duct injury: 1) as shown in Table 2. Port site hematoma was managed by evacuation, port site SSI was managed by dressing and antibiotic coverage as per culture sensitivity, and serosanguineous collection in GB fossa was managed by image guided aspiration under antibiotic coverage. The one in which common bile duct (CBD) injury occurred was managed by conversion to open procedure by the experienced surgeon, and hepaticojejunostomy was performed.

Hence, incidence of bile duct injury (BDI) in our study was 1 in 230 cases (0.43%).

## 6. Discussion

This Rouviere's sulcus is seen very clearly during laparoscopic cholecystectomy due to the pressure of CO_2_ insufflation opening up the sulcus widely and due to the enhanced illumination and image quality of the laparoscopic camera.

### 6.1. Age Distribution

The mean age of patients in our study was 41.56 ± 14.27 years, suggesting that uncomplicated gall stone is a disease of middle age population. This is in accordance with the study by Sachdeva et al. in which the mean age was 41.5 ± 15.4 years [6].

The majority in our study were in the range of 26–35 years of age. This suggests a shift in the trend of gall stone disease from middle to young age, and this is in accordance with the study by Gupta et al. [7].

### 6.2. Gender Distribution

In our study, we observed that females had slightly higher preponderance of gall stone with female to male ratio of 2.43 : 1. In a study by Sachdeva et al., female had higher preponderance of gall stone with female to male ratio of 1.56 : 1 [6].

The sex hormones are believed to place females at higher risk. Estrogen increases biliary cholesterol secretion causing supersaturation of bile.

### 6.3. RS Characteristics

RS was present in around 90% of the patients, and the commonest one was the open sulcus type followed by scar type, closed sulcus type, and slit type RS, respectively. Dahmane et al. reported that Rouviere's sulcus was present in 82% of normal livers, and open RS was the commonest [5]. Reynaud et al. and Hugh et al. noted RS in 73% and 78% of livers, respectively [2, 4].

In our study, RS was oblique to the anterior, inferior, and external edge of the liver in 123 (59.1%) cases, while in 85 (40.9%) cases, it was transverse. In the study by Dahmane et al., the sulcus was oblique to the anterior, inferior, and external edge of the liver in 97% of cases and horizontal in 3% [5].

The orientation of RS as oblique or horizontal seen during laparoscopic cholecystectomy may change with orientation of camera and hence can be interpreted differently by different operating surgeons. Hence, this could be subject to bias.

### 6.4. Operative Time and RS

The operative time in RS visible group was reduced by around 25% in comparison to RS not visible group. In RS visible group, no time would have been spent in adhesion release, and the operating surgeons might not had any preoccupied hesitancy regarding chance of CBD injury as RS marks the plane of CBD.

### 6.5. Duration of Hospital Stay and RS

The duration of hospital stay in the RS visible was significantly less than that in RS not visible group. The shorter hospital stay in RS visible group could be attributed to lesser operative time, lesser dissection, and lesser need to place drain in this group. Majority of patients with drain had tendency toward going home only after removal of drain.

### 6.6. Intra/Postoperative Complications and RS

1 *minor* complication occurred in each group: RS visible initially group and RS visible on adhesion release group. On the other hand, 3 *minor* complications occurred in RS not visible group. Only one major complication (bile duct injury) occurred in RS not visible group.

Even though this discrepancy on occurrence of complication in various groups was statistically not significant because of small sample size, it could be clinically significant and it can be concluded that recognition of RS before dividing cystic artery/duct during laparoscopic cholecystectomy avoids *major* complications.

Peti and Moser described a case where identification of the sulcus helped to prevent common bile duct injury [8]. Hugh et al. and Zubair et al. had shown minimal common bile duct injury during laparoscopic cholecystectomy by beginning the dissection ventral to the RS [4, 9].

The overall incidence of bile duct injury (BDI) in our study was 1 in 230 cases, which amounts to 0.4%. Flum et al. reported that the incidence of bile duct injury increased from 0.2% in open cholecystectomy era to 0.5% after laparoscopic cholecystectomy [10].

## 7. Summary

The RS was present in 90.4% of the cases, and the open sulcus type was the commonest (54%), followed by the scar type (22.9%), the closed sulcus type (12.5%), and the slit type (10.6%), respectively. In 59.1% of the cases, it was oblique to the anterior, inferior, and external edge of the liver, and in 40.9% of the cases, it was transverse. The mean ± SD values for operative time and duration of hospital stay in the RS visible and the RS not visible groups were 29.16 ± 8.736 and 42.9 ± 23.646 minutes, and 1.26 ± 0.440 and 1.90 ± 0.910 days, respectively (*p* value ≤0.001). One minor complication occurred in each group: RS visible initially group and RS visible on adhesion release group. On the other hand, 3 minor complications occurred in RS not visible group. Only one major complication occurred in RS not visible group.

## 8. Conclusion

Rouviere's sulcus is present in majority of population in our part of the world, and open sulcus type is the commonest. Recognition of RS before dividing cystic artery/duct during laparoscopic cholecystectomy avoids major complications and is associated with short operative time as well as short duration of hospital stay.

### 8.1. Limitation

This study had few limitations like small sample size, short term follow-up, observational bias, and being single center-based study.

### 8.2. Recommendation

Identification of RS is a predictor of safe laparoscopic cholecystectomy.

## Figures and Tables

**Table 1:** Correlation of operative time and duration of hospital stay with RS.

	RS visible	RS not visible	*t*-test	*p* value
Operative time (minute), mean ± SD	29.16 ± 8.736	42.9 ± 23.646	6.432	<0.001
Duration of hospital stay (day), mean ± SD	1.26 ± 0.440	1.90 ± 0.910	4.86	<0.001

**Table 2:** Intra/postoperative complications and RS.

Complications	RS visible before dividing cystic artery/duct dissection group, *n* = 208		RS not visible before dividing cystic artery/duct, *n* = 22 (absence of RS)
	RS visible initially, *n* = 162	RS visible on release of adhesion, *n* = 46	
Major	0	0	1
(i) Common bile duct injury			

Minor	0	0	1
(i) Port site SSI	1	0	1
(ii) Port site hematoma	0	1	1
(iii) Serosanguineous collection in GB fossa			

Total	2		4

## Data Availability

The data used to support the findings of the study can be made available from the corresponding author upon request.

## Additional Points

*Highlights.* Well established: Rouviere's sulcus is present in 70-80% of population and is an important anatomical landmark as the common bile duct lies below its level. Newly established: Rouviere's sulcus is present in 90% population in our part of world and open sulcus type is the commonest one. Recognition of RS before dividing cystic artery/duct during laparoscopic cholecystectomy avoids major complication and is associated with short operative time as well as short duration of hospital stay.

## References

[1] Couinaud C. (1994). The paracaval segments of the liver. *Journal of Hepato-Biliary-Pancreatic Surgery*.

[2] Reynaud J. A., Brack A., Grivet J. P., Trudelle Y. (1991). Interaction of phospholipid vesicles with basic amphiphilic polypeptides. *Peptides 1990*.

[3] Gans (1995). Study of anatomy of the intrahepatic structures and its repercussions of hepatic surgery. *Ph.D. thesis*.

[4] Hugh T. B., Kelly M. D., Mekisic A. (1997). Rouvière’s sulcus: a useful landmark in laparoscopic cholecystectomy. *British Journal of Surgery*.

[5] Dahmane R., Morjane A., Starc A. (2013). Anatomy and surgical relevance of Rouviere’s sulcus. *Scientific World Journal*.

[6] Sachdeva S., Ansari M., Anees A., Khan Z., Khalique N. (2011). Lifestyle and gallstone disease: scope for primary prevention. *Indian Journal of Community Medicine*.

[7] Gupta R. L., Sharma S. B., Kumar S. P. (1998). Changing trends in gall bladder stone disease- an observation. *Indian Journal of Medical Sciences*.

[8] Peti N., Moser M. A. J. (2012). Graphic reminder of Rouviere’s sulcus: a useful landmark in cholecystectomy. *ANZ Journal of Surgery*.

[9] Zubair M., Habib L., Memon F. (2009). Rouviere’s sulcus: a guide to safe dissection and laparoscopic cholecystectomy. *Pakistan Journal Of Surgery*.

[10] Flum D. R., Cheadle A., Prela C., Dellinger E. P., Chan L. (2003). Bile duct injury during cholecystectomy and survival in medicare beneficiaries. *JAMA*.

